# Protein interaction patterns in different cellular environments are revealed by in-cell NMR

**DOI:** 10.1038/srep14456

**Published:** 2015-09-24

**Authors:** Letizia Barbieri, Enrico Luchinat, Lucia Banci

**Affiliations:** 1 Magnetic Resonance Center - CERM, University of Florence, Via Luigi Sacconi 6, 50019 Sesto Fiorentino, Florence, Italy; 2 Giotto Biotech S.r.l., Via Madonna del Piano 6, 50019 Sesto Fiorentino, Florence, Italy; 3 Department of Biomedical, Clinical and Experimental Sciences, University of Florence, Viale Morgagni 50, 50134 Florence, Italy; 4 Department of Chemistry, University of Florence, Via della Lastruccia 3, 50019 Sesto Fiorentino, Florence, Italy

## Abstract

In-cell NMR allows obtaining atomic-level information on biological macromolecules in their physiological environment. Soluble proteins may interact with the cellular environment in different ways: either specifically, with their functional partners, or non-specifically, with other cellular components. Such behaviour often causes the disappearance of the NMR signals. Here we show that by introducing mutations on the human protein profilin 1, used here as a test case, the in-cell NMR signals can be recovered. In human cells both specific and non-specific interactions are present, while in bacterial cells only the effect of non-specific interactions is observed. By comparing the NMR signal recovery pattern in human and bacterial cells, the relative contribution of each type of interaction can be assessed. This strategy allows detecting solution in-cell NMR spectra of soluble proteins without altering their fold, thus extending the applicability of in-cell NMR to a wider range of proteins.

In-cell NMR is a relatively recent application of NMR spectroscopy, which has the ability to acquire structural and functional information on biomolecules in the native environment of living cells, with atomic resolution^1^,^2^. This ability places in-cell NMR in a unique position, combining a structural biology technique with the full complexity of the cellular environment, including its pH and redox properties, macromolecular crowding and possibly the presence of the relevant interaction partners. To date, most of the in-cell NMR applications have focused on proteins located in the cytoplasm of prokaryotic and eukaryotic cells, through solution NMR experiments^3^,^4^,^5^,^6^,^7^,^8^,^9^,^10^. It has been observed in a number of cases that proteins are invisible to in-cell solution NMR, as their signals are broadened beyond detection^11^,^12^,^13^,^14^,^15^. This effect cannot be a consequence of the increased viscosity of cellular environments compared to diluted buffer solutions, which is modest in both bacterial and eukaryotic cells^16^,^17^. Such NMR signal broadening has been attributed to weak, non-specific interactions occurring between the protein of interest and large molecular assemblies or cellular structures such as membranes, nucleic acids and protein complexes, which would slow down the average molecular tumbling rate, leading to increased spin relaxation rates and signal loss^12^,^13^,^18^,^19^. This layer of weak interactions between soluble proteins and other cellular components, the so-called “quinary structure”^20^, has been hypothesized to influence the physiological behaviour of intracellular proteins, and is recently being explored by NMR^21^,^22^. In addition, functional interactions with the physiological partners can contribute to signal broadening^15^. Such specific interactions are expected to contribute less to the signal broadening than non-specific interactions, as the observed protein is usually present at much higher levels than its physiological partner(s), thus all the binding sites of the latter are saturated and the remaining free protein is observed. In practice however, it is difficult to assess the relative contribution of these different types of interactions, as each of them is a function of relative amount of partner(s), of the binding rates and affinity, and of the tumbling rates of the complexes. To investigate the loss of NMR signals due to interactions, point mutations can be introduced on the protein of interest, in order to change its surface properties (i.e. electrostatic potential, hydrophobicity, size) and binding affinities. The NMR signal recovery can then be used to assess the overall ‘stickiness’ of the protein surface, although no information on the nature of the interaction is obtained.

If the protein of interest is observed in different cellular environments, the changes in surface properties may impact differently on the NMR signal recovery. For example, a human protein observed in human cells may interact both with its functional partners, and with other cellular components. When the same protein is observed in a non-native environment, such as a bacterial cell, some or all functional partners will be absent, and only the non-specific interactions will occur. By analyzing the NMR signal recovery of the protein mutated on the potential interaction regions in both cellular environments, information can be obtained on the relative contribution of each type of interaction on the protein mobility.

As a test system, we investigated through in-cell NMR the interaction properties of the human protein profilin 1 (PFN1) in the cytoplasm of both human and bacterial cells. PFN1 is a small globular cytoskeletal protein involved in a complex network of molecular interactions^23^. PFN1 is expressed at high levels in all tissues, and exerts a major role in actin remodelling^24^. It interacts with G-actin monomers to form a 1:1 complex and promotes actin polymerization at the barbed end of the F-actin filament^25^,^26^. This mechanism is regulated through an increase in phosphatidylinositol (4,5)-bisphosphate [PtdIns(4,5)P_2_] concentration, which also binds to PFN1 and modulates profilin-actin complex formation^27^,^28^,^29^. Among the phosphoinositide lipids, PtdIns(4,5)P_2_ is the most abundant and has an effective cellular concentration of approximately 10 μM in mammalian cells^30^,^31^. Additionally, PFN1 has been shown to interact with a large number of proteins that contain poly-L-proline (PLP) motifs^32^,^33^,^34^. Profilin1 interaction with PLP motifs is unusually tight and has a role in the cytoskeletal remodeling and vesicle trafficking. The PLP binding site on profilin1 is distinct from the actin-binding site, and both sites have only partial overlap with the phosphoinositides binding region^23^. These binding sites have been extensively characterized *in vitro*, making PFN1 an ideal test case for analyzing its specific and non-specific interactions with the cellular environment. As expected, wild-type (WT) PFN1 was found to be invisible by in-cell NMR both in bacterial and human cells. We introduced a series of mutations known to affect each type of specific interaction (i.e. actin, PtdIns(4,5)P_2_ and PLP), so to eliminate each interaction individually or in combination, and we analyzed their effects on PFN1 by in-cell NMR. When all three types of mutations were combined together, the protein could be detected in both bacterial and human cells. However, the partial mutants showed a different behaviour in the two cellular environments, and allowed discriminating between functional and non-specific interactions.

## Results and Discussion

### Profilin 1 interacts both specifically and non-specifically in the cytoplasm

**Figure 1:**
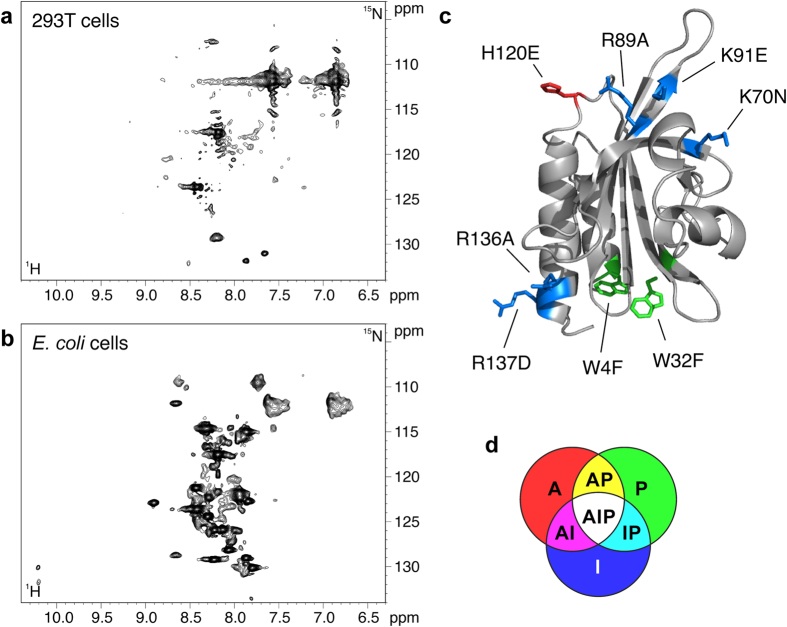
^**1**^**H-**^**15**^**N SOFAST-HMQC spectra of WT PFN1 in human (a) and E. coli cells (b) see** Fig. S1 **for the cellular localization of WT PFN1.** (**c**) cartoon representation of the structure of WT PFN1; the mutated residues are labelled and color-coded based on the type of interaction (actin in red, PtdInsP_2_ in blue, PLP in green); (**d**) Diagram showing the additive combinations of the three types of mutations.

Wild type human PFN1 was overexpressed in HEK293T and *E. coli* cells. The protein was mainly localized in the cytosol of human cells, as determined by SDS-PAGE (Supplementary Fig. 1). The amide signals of U-^15^N WT PFN1 were not observed in both human and bacterial cells, indicating that the occurrence of interactions in both cellular environments decrease the tumbling rate of PFN1 and signal broadening beyond detection (Fig. 1a,b). Strong, well resolved amide signals were detected upon cell lysis (Supplementary Fig. 2a), indicating that the protein is present at high levels in the cells and that most interactions are lost upon breaking the cellular structure. The protein is in a folded state, and the amide chemical shifts of the backbone compare well to those reported by Metzler and co-workers^35^, with the exception of few chemical shifts which were re-assigned on the U-^13^C,^15^N labelled protein present in a bacterial lysate (Supplementary Fig. 3a). The lack of NMR signals in the human cell spectrum was expected, as PFN1 is continuously engaged in several functional interactions inside the cell. However, the same effect was observed in bacterial cells, indicating that PFN1 is also interacting non-specifically with cellular components present in *E. coli* and, presumably, also in human cells.

### *Combined surface mutations make PFN1 detectable* in human cells

In order to assess the contribution of the various interactions of PFN1 with the environment, we introduced a series of mutations on surface residues known to be involved in the functional interactions of PFN1, based on the information available in the literature (Fig. 1c). For each of the three distinct interaction surfaces of PFN1, one or more residues were mutated: H120E^36^ on the actin interaction surface (“A” mutant); W4F and W32F^32^ on the PLP interaction surface (“P” mutants); K70N, R89A, K91E, R136A, R137D^29^ on the PtdInsP_2_ interaction surface (“I” mutants). These mutations were then combined together, to abolish more types of interactions simultaneously (Fig. 1d and Table 1). By comparing the NMR properties of each mutant in bacterial and human cells, we evaluated the contribution of each interaction surface on the overall tumbling of the protein in the two cellular environments.

**Figure 2:**
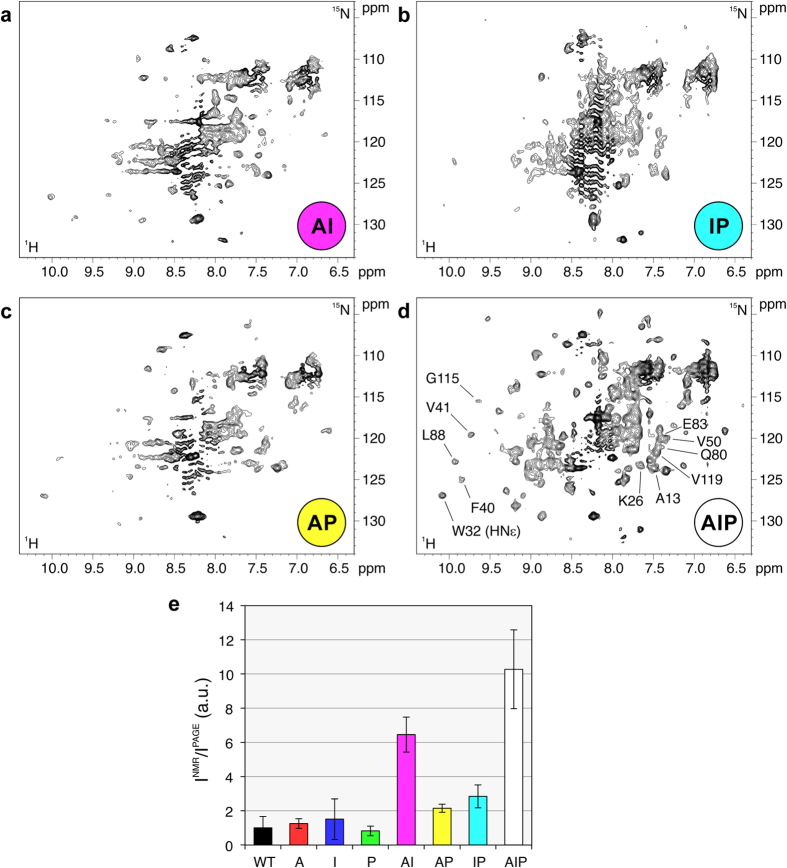
^1^H-^15^N SOFAST-HMQC spectra of human cells expressing double-type PFN1 mutants “AI” (a) “IP” (b) “AP” (c) and the triple-type PFN1 mutant “AIP” (d). The mutant types are color-coded as in the diagram shown in Fig. 1d. The corresponding human cell lysates are shown in Fig. S2. The crosspeaks used to estimate the fraction of visible protein over the total protein for each mutant are shown in (**d**), labelled according to the assignment. (**e**) Ratios between the signal intensity of the ^1^H-^15^N SOFAST-HMQC spectra and the normalized band intensity of a Coomassie-stained SDS-PAGE of lysates of human cells expressing each PFN1 mutant. The error bars are calculated from the uncertainty on the NMR signal intensities (calculated as standard deviation of the spectral noise).

In human cells, the ^1^H-^15^N NMR spectra of single-type PFN1 mutants (“A”, “I”, “P”) displayed almost no signals, indicating that the inhibition of only one type of interaction is not sufficient to make the protein free to tumble, and the protein is still engaged with other interacting partners. When two out of three types of interactions were abolished (“AI”, “AP”, “IP”), a few weak, resolved crosspeaks could be detected in the in-cell ^1^H-^15^N NMR spectra of all three double-type mutants (Fig. 2a–c). When all three types of mutations were introduced (“AIP” mutant), PFN1 was clearly detected in the in-cell ^1^H-^15^N NMR spectrum, confirming that when all types of interactions are abolished the protein is mostly in the free-tumbling state (Fig. 2d). For each mutant, the cell lysates were analyzed by NMR, and they always showed well resolved signals which, except for the mutation sites, compare very well with those of the WT (Supplementary Fig. 2b–h and Supplementary Fig. 3). This indicates that the mutations do not affect the folding state and the conformation of PFN1.

In order to estimate the fraction of free protein over the total protein, a subset of crosspeaks in the in-cell ^1^H-^15^N NMR spectra of each mutant were integrated (shown in Fig. 2d), and the average intensity was normalized by the total level of intracellular protein measured by SDS-PAGE (ranging from 170 ± 40 μM for the “A” mutant to 60 ± 15 μM for the “AIP” mutant, Supplementary Fig. 4a,c). The obtained data show that, assuming all the “AIP” mutant protein to be free in the cytoplasm, WT and single-type PFN1 mutants are for the most part engaged in some kind of interaction, while ~1/4 of the total “AP” and “IP” mutants and ~2/3 of the “AI” mutant are not interacting (Fig. 2e). To better understand the nature of the interactions, the 1D ^1^H NMR spectra of human cells were also analyzed. Unlike ^1^H-^15^N correlation NMR spectra, which only detect the free protein, a 1D ^1^H NMR experiment should still detect aliphatic ^1^H signals of the protein engaged in soluble complexes (i.e. not associated with the membrane or the cytoskeleton), thanks to the higher S/N ratio, no chemical exchange with the solvent and presence of side-chain internal motions. The peak intensities in the aliphatic region of the ^1^H in-cell NMR spectra (Supplementary Fig. 5a) were integrated for each PFN1 mutant and normalized by the total protein level. The obtained values show that, compared to WT PFN1, the fraction of detectable protein remains unchanged for the proteins containing “A” and/or “P” mutations, and increases for all the mutants which contained the “I” mutations, including those still largely invisible by ^1^H-^15^N NMR (Supplementary Fig. 5b). Although these data can only be interpreted qualitatively, as the NMR relaxation properties depend also on the exchange rates between free and bound states, and on the molecular size of the latter, they indicate that PFN1 interacts with non-soluble cellular components mainly through the PtdInsP_2_-binding surface. The intracellular content of PtdIns(4,5)P_2_ is significantly lower (around 10 μM) compared to the expression levels of the PFN1 mutants (in the order of 100 μM), thus the interaction of PFN1 with PtdIns(4,5)P_2_ alone is unlikely to explain the effects observed in the ^1^H NMR spectra. Therefore, the observed effect likely arises from non-specific interactions of PFN1 through the PtdInsP_2_-binding surface. When the “I” type mutations are introduced, a larger fraction of PFN1 is detected in the ^1^H NMR spectra, but *not* in the ^1^H-^15^N NMR spectra, suggesting that PFN1 is still involved in interactions with soluble partners through the actin- and PLP-binding surfaces. Among the possible interacting proteins, actin is present in the cytoplasm at concentrations ranging between 100 and 1000 μM in eukaryotic cells^37^,^38^, a fraction of which is soluble G-actin. Therefore, PFN1 likely interacts with the functional partner through the actin-binding site. Concerning PLP-binding, the aromatic to aromatic substitution W4F is sufficient to make the “AI” mutant (almost undetected) clearly detectable (“AIP”) by ^1^H-^15^N NMR. This observation strongly suggests that also the PLP-binding surface interacts mostly with specific partners in human cells.

### Non-specific interactions of PFN1 in bacterial cells

**Figure 3:**
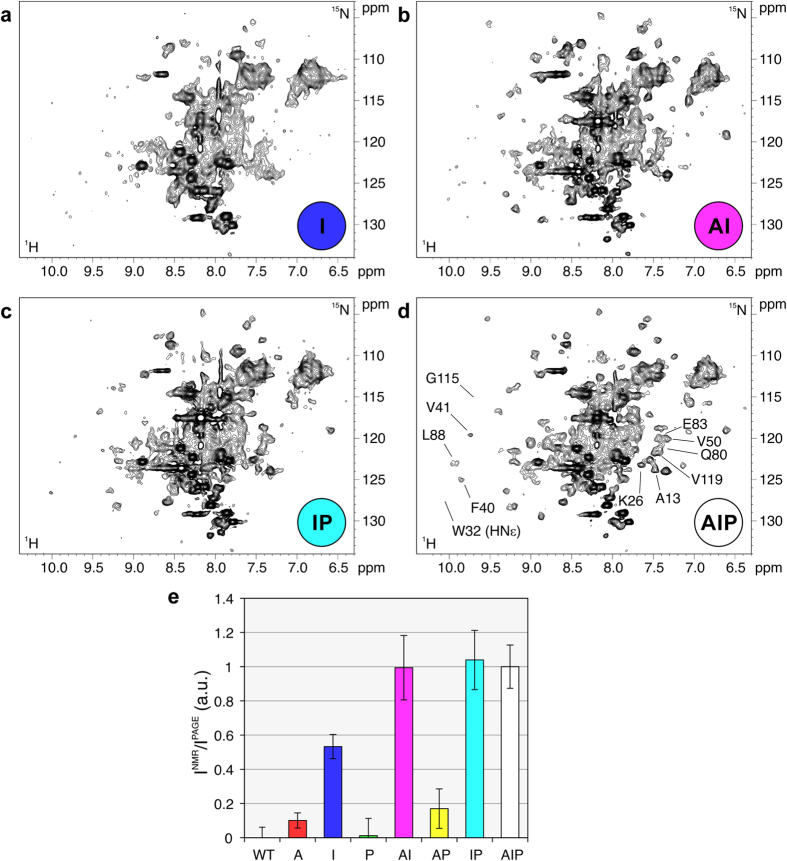
^1^H-^15^N SOFAST-HMQC spectra of *E. coli* cells expressing the single-type PFN1 mutant “I” (a) double type PFN1 mutants “AI” (b) “IP” (c) and the triple-type PFN1 mutant “AIP” (d). The mutant types are color-coded as in the diagram shown in Fig. 1d. The crosspeaks used to estimate the fraction of visible protein over the total protein for each mutant are shown in (**d**) labelled according to the assignment. (**e**) Ratios between the signal intensity of the ^1^H-^15^N SOFAST-HMQC spectra and the normalized band intensity of a Coomassie-stained SDS-PAGE of *E. coli* lysates expressing each PFN1 mutant. The error bars are calculated from the uncertainty on the NMR signal intensities (calculated as standard deviation of the spectral noise).

**Figure 4:**
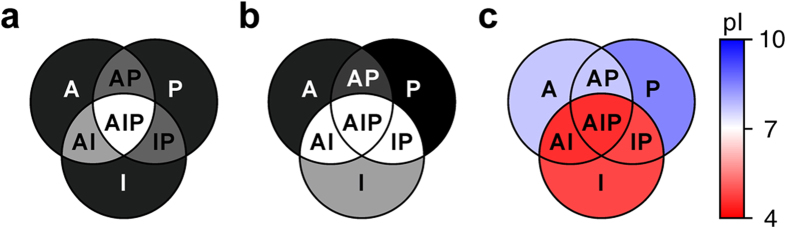
Diagrams summarizing the different behaviour of the PFN1 mutants in human (a) and bacterial cells (b), as observed by in-cell ^1^H-^15^N SOFAST-HMQC. The greyscale is qualitatively proportional to the signal intensity, from dark grey (no signals) to white (well detected signals); (**c**) diagram showing the differences in isoelectric point (pI) between the PFN1 mutants (see Table 1), color-coded in red-white-blue.

Bacterial cells lack most of the known partners of PFN1. Phosphoinositides are not reported in Gram-negative bacteria^39^; the only actin structural homologue known in *E. coli*, MreB, has very low sequence identity^40^ and lacks loops responsible for actin-binding proteins recognition^41^; poly-L-proline motifs are encoded in bacterial genomes, but are not known to function as ubiquitous recognition motifs as in eukaryotes. Therefore, in bacterial cells human PFN1 can be assumed to engage in non-specific interactions only. In bacteria, the surface mutations had interestingly different effects on the NMR spectra of intracellular PFN1, compared to human cells. Two single-type PFN1 mutants, “A” and “P”, did not give rise to any signal in the ^1^H-^15^N in-cell NMR spectra, like in human cells. However, the single-type “I” mutant, which is not detectable in human cells, can be detected, although barely, in the bacterial in-cell NMR spectrum (Fig. 3a). The double-type mutants “AI and “IP” gave rise to clearly detectable signals (Fig. 3b,c), whereas the “AP” mutant was still completely invisible by ^1^H-^15^N in-cell NMR. Consistently, the triple-type mutant “AIP” was also detected in bacterial cells, and gave rise to detectable signals in the ^1^H-^15^N NMR spectrum similar to the “AI” and “IP” PFN1 mutants (Fig. 3d). The fraction of free protein over the total protein was estimated in bacterial cells, as done for human cells, by integrating a subset of crosspeaks in the in-cell ^1^H-^15^N NMR spectra of each mutant (shown in Fig. 3d), and normalizing by the total level of intracellular protein (ranging from 300 ± 100 μM for WT PFN1 to 100 ± 30 μM for the “AP” mutant, Supplementary Fig. 4b). The resulting data (Fig. 3e) indicate that the non-specific interactions of PFN1 with components of the bacterial cell involve principally the PtdInsP_2_ interaction surface, while the actin- and PLP-binding surfaces give a lesser contribution to the overall behaviour of the protein in the bacterial cytoplasm. The data obtained in bacteria by ^1^H-^15^N NMR (Fig. 3e) show the same trend observed by ^1^H NMR in human cells (Supplementary Fig. 5), confirming that PFN1 is involved in non-specific interactions through the PtdInsP_2_-binding surface in both cellular environments. The different behaviour of the PFN1 mutants in human and bacterial cells, observed by ^1^H-^15^N NMR, is qualitatively summarized in Fig. 4a,b, and show that the actin- and PLP-binding surfaces contribute less to the interactions of PFN1 in bacteria than in human cells.

### PFN1 likely interacts non-specifically within the cell by electrostatic interactions

Among the mutations introduced, those which affect the PtdInsP_2_ interaction have the largest impact on the surface charge distribution, as lysine and arginine residues are replaced either with neutral (K70N, R89A, R136A) or with negatively charged residues (K91E, R137D). The effect of these substitutions is reflected by a net decrease of the protein isoelectric point (pI), from 8.4 (WT) to 4.8 (“I^5x^” mutant) (Table 1). It has been previously shown that proteins with positive charged patches surface on the surface can interact with negatively charged constituents of the bacterial cell, such as the polar head groups of the internal membrane and nucleic acids^18^,^19^,^22^. Moreover, charge-inversion mutations have been reported to affect protein stability within the bacterial cell more than in dilute buffer^21^. The interaction of PFN1 with bacterial components was further investigated in bacterial lysates. At room temperature, WT PFN1 still experienced interactions after cell lysis, giving rise to much weaker signals in the ^1^H-^15^N NMR spectrum compared to the “I” PFN1 mutant (Supplementary Fig. 6a,b). Addition of RNase A and MgCl_2_ to the lysates partially restored the signals of WT PFN1, while the “I” mutant was unaffected (Supplementary Fig. 6c,d). Therefore, the occurrence of interactions with RNA may provide a simple explanation for the undetectability of NMR signals of WT PFN1 in the bacterial cytoplasm, and would also explain the increased detectability of the PFN1 mutants with lower pI (i.e. “I”, “AI”, “IP” and “AIP”, Fig. 4c). In summary, the different patterns of ^1^H-^15^N NMR signal recovery of the PFN1 mutants obtained in human and bacterial cells indicate that, while non-specific electrostatic interactions are abundant in both environments, functional interactions also contribute to the ^1^H-^15^N NMR signal loss in human cells due to the formation of complexes with soluble partners which are absent in bacteria.

## Conclusions

Recent applications of in-cell NMR have shown that soluble cytoplasmic proteins are often engaged in many weak, non-specific interactions within the cell, which in some cases lead to the complete loss of NMR signals, preventing further analysis. The same proteins, when placed in their physiological environment, also interact with their specific partners, and many of these interactions have already been identified and characterized *in vitro*, as is the case of PFN1. Here we showed that the contribution of these different kinds of interactions can be investigated by introducing mutations on the appropriate surface residues, and by comparing the in-cell NMR behaviour of each mutant in different cellular environments: the real physiological location (e.g. the human cytoplasm) and a model environment in which the specific partners are absent, and only the effects of non-specific interactions are observed (e.g. the bacterial cytoplasm). In principle, this approach can be extended to any other soluble protein for which the interactions with physiological partners have been identified and characterized, provided that the partners are absent in the bacterial cells. Proteins which are conserved both in prokaryotes and higher organisms may not be suitable for this kind of approach, as functional-like interactions could still occur between the investigated protein and the functional partners of its bacterial homolog.

From a methodological standpoint, the mutational approach described here expands the range of applicability of in-cell NMR, as it can be adapted to other proteins of choice whenever the wild-type is not detectable by solution in-cell NMR. With some *a priori* knowledge, surface mutants can be designed to specifically affect a subset of interactions, either non-specific or with selected partners, in order to recover the protein signals with minimal alterations of the protein fold. NMR signal recovery will thus allow investigating by in-cell NMR protein/ligand interactions, cofactor binding and post-translational modifications. In principle, NMR methods suited for the detection of slow-tumbling molecules^42^,^43^,^44^,^22^ can also be combined with this approach to further increase the size limit, therefore allowing in-cell NMR analysis of larger proteins, or proteins in complex with soluble partners.

## Methods

The synthetic gene of WT PFN1 was purchased from Eurofins Genomics. The DNA was cloned in the pHLsec vector^45^ between EcoRI and XhoI restriction sites and in the pET20b(+) vector (Novagen) between NdeI and XhoI restriction sites. All the mutants were obtained by site-directed mutagenesis on the WT gene inside the pJET1.2/blunt vector (Thermo Scientific). The mutated genes were sub-cloned in the pHLsec vector between EcoRI and XhoI restriction sites and in pET20b(+) or pET21a(+) vectors (Novagen) between NdeI and XhoI restriction sites. All clones were verified by DNA sequencing. The isoelectric point was calculated for each mutant with the ProtParam tool on the ExPASy server^46^.

HEK293T cells were maintained in DMEM (high glucose, D6546, Sigma) supplemented with L-glutamine, antibiotics (penicillin and streptomycin) and 10% FBS (Gibco) in uncoated 75-cm^2^ plastic flasks, and were grown at 37 °C, 5% CO_2_ in a humidified atmosphere. The cells were transiently transfected with the pHLsec plasmid containing the WT or mutated PFN1 gene using polyethylenimine (PEI), as described elsewhere^7^,^45^. Commercial DMEM medium was used for unlabelled in-cell NMR samples; BioExpress6000 medium (Cambridge Isotope Laboratories) was used for U-^15^N labelling. Expression medium was supplemented with 2% FBS and antibiotics. Samples for in-cell NMR were prepared following a reported protocol^7^: transfected HEK293T cells were detached with trypsin, suspended in DMEM + 10% FBS, washed once with phosphate-buffered saline and re-suspended in one pellet volume of DMEM supplemented with 90 mM glucose, 70 mM HEPES for increased pH stability and 20% D_2_O. The cell suspension was transferred in a 3 mm Shigemi NMR tube and cells were allowed to settle on the bottom. Cell viability before and after NMR experiments was assessed by trypan blue staining. After the NMR experiments, the cells were lysed by the freeze-thaw method after being suspended in one pellet volume of PBS buffer supplemented with 0.5 mM EDTA and 4-(2-aminoethyl)-benzenesulfonyl fluoride hydrochloride (AEBSF). The lysate was centrifuged at 16,000 × *g* for 1 hour at 4 °C, and the supernatant was collected for NMR analysis in a standard 3 mm NMR tube.

U-^15^N labelled samples for in-cell NMR in *E. Coli* cells were prepared as follows: a cell culture of BL21(DE3) Gold transformed with a pET20b(+) or pET21a(+) plasmid containing the WT or mutated PFN1 gene, was grown overnight at 30 °C in 35 mL of LB medium. After gentle centrifugation (3000 g) for 20 minutes, the cells were re-suspended in 50 mL of M9 minimal medium [M9 buffer (7 g/L K_2_HPO_4_, 3 g/L KH_2_PO_4_, 0.5 g/L NaCl, pH 7.4) supplemented with 2 mM MgSO_4_, 0.1 mM CaCl_2_, 1 mg/L biotin, 1 mg/L thiamine, antibiotic] containing 1 g/L (^15^NH_4_)_2_SO_4_ and 3 g/L of unlabelled glucose to obtain an OD_600_ of ~1.6. After 10 min recovery time, overexpression was induced with 0.5 mM IPTG, and carried out at 30 °C for 4 h. The cells were washed once with 50 mL of M9 buffer in order to remove nutrients, metal ions and any excreted by-product, and they were harvested through gentle centrifugation. The pellet was then re-suspended in M9 buffer until 500 μL of a ~50% v./v. cell slurry were obtained. 50 μL of D_2_O were added, and the final volume was put in a 5 mm NMR tube. Cleared cell lysates for in-cell NMR experiments were prepared as follows: after removal of the supernatant, the cell pellet was re-suspended in an equal volume of M9 buffer. The cells were then lysed by ultrasonication. Then the lysate was centrifuged at 18000 g for 40 minutes, the supernatant was collected and its volume was brought to 500 μL with M9 buffer. 50 μL of D_2_O were then added.

NMR experiments on human cells and lysates were acquired at a 950 MHz Bruker Avance spectrometer equipped with a TCI CryoProbe. NMR experiments on bacterial cells and lysates were acquired at a 900 MHz Bruker Avance spectrometer equipped with a TCI CryoProbe. 1D ^1^H (zgesgp Bruker pulse sequence) and 2D ^1^H-^15^N SOFAST HMQC spectra^47^ were acquired at 308 K unless otherwise specified. The total acquisition time for each cell sample was about 1 hour. The supernatant of each cell sample was checked in the same experimental conditions to exclude protein leakage. The same NMR spectra were also acquired on the cell lysates. All the spectra were processed with Bruker Topspin software. The NMR spectra acquired on human cells and lysates were further processed by subtracting a spectrum of untransfected cells/cell lysate acquired in the same experimental conditions and identically processed, to eliminate the signals arising from unspecific ^15^N labelling of the cells. The sequential backbone assignment of WT PFN1 and “AIP” mutant PFN1 was obtained using CARA software, from 3D HNCO, HN(CA)CO, HNCA, CBCA(CO)NH experiments acquired at 308 K on bacterial cell lysates containing ^13^C,^15^N-labelled proteins.

For the RNA interaction experiments, bacterial cells lysates were prepared in PBS buffer, and analyzed by NMR at 296 K. Few crystals of RNase A were then added together with MgCl_2_ (to a final concentration of 35 mM), and the samples were incubated for 30′ at 293 K followed by NMR analysis.

## Additional Information

**How to cite this article**: Barbieri, L. *et al.* Protein interaction patterns in different cellular environments are revealed by in-cell NMR. *Sci. Rep.* **5**, 14456; doi: 10.1038/srep14456 (2015).

## Supplementary Material

Supplementary Information

## Figures and Tables

**Table 1:**
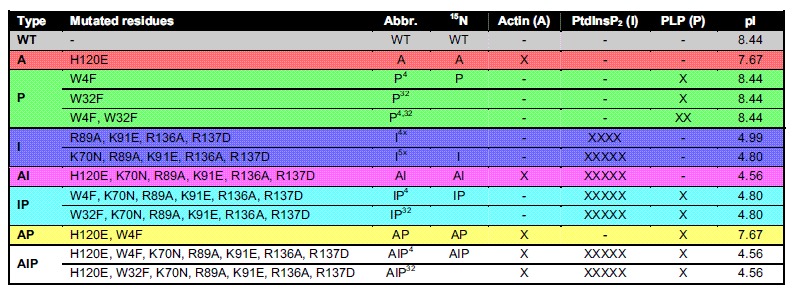
List of all the mutations introduced in the PFN1 amino acid sequence.

For each combination, the abbreviated name and the number of mutated residues for each type of interaction surface are indicated (X), and the calculated isoelectric point (pI) is reported. Specific mutants of each type for which ^1^H-^15^N NMR spectra were recorded are indicated. The mutant types are color-coded as in the diagram shown in Fig. 1d.
