# Supplementary material for: Protein interaction patterns in different cellular environments are revealed by in-cell NMR
Source: Sci Rep. 2015 Sep 24;5:14456. doi: 10.1038/srep14456 (PMC4585868; doi:10.1038/srep14456)
Supplement: Supplementary Information [file srep14456-s1.doc]

Supplementary Information

**Protein interaction patterns in different cellular environments are revealed by in-cell NMR**

Letizia Barbieri^1,2^, Enrico Luchinat^1,3^, Lucia Banci^1,4,*^

^1^Magnetic Resonance Center - CERM, University of Florence, Via Luigi Sacconi 6, 50019 Sesto Fiorentino, Florence, Italy

^2^Giotto Biotech S.r.l., Via Madonna del Piano 6, 50019 Sesto Fiorentino, Florence, Italy.

^3^Department of Biomedical, Clinical and Experimental Sciences, University of Florence, Viale Morgagni 50, 50134 Florence, Italy

^4^Department of Chemistry, University of Florence, Via della Lastruccia 3, 50019 Sesto Fiorentino, Florence, Italy

**Supplementary Figures**


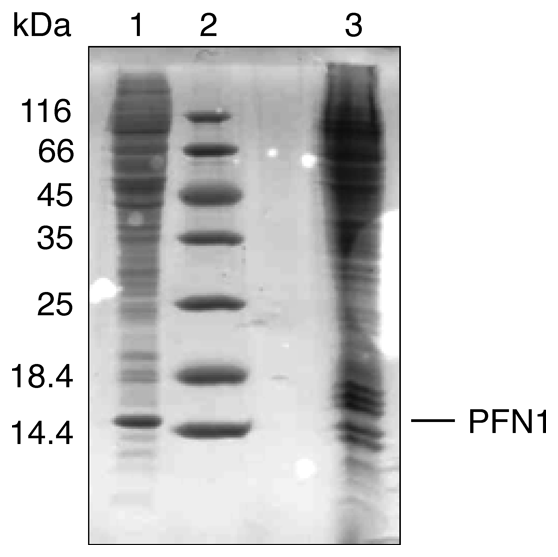


**Supplementary Figure 1.** Subcellular localization of WT PFN1. A band corresponding to overexpressed PFN1 is only detected in the cytoplasmic extract (lane 1), and not in the fraction containing the nuclei and other organelles (lane 3). The molecular weights of the protein marker (lane 2) are indicated.


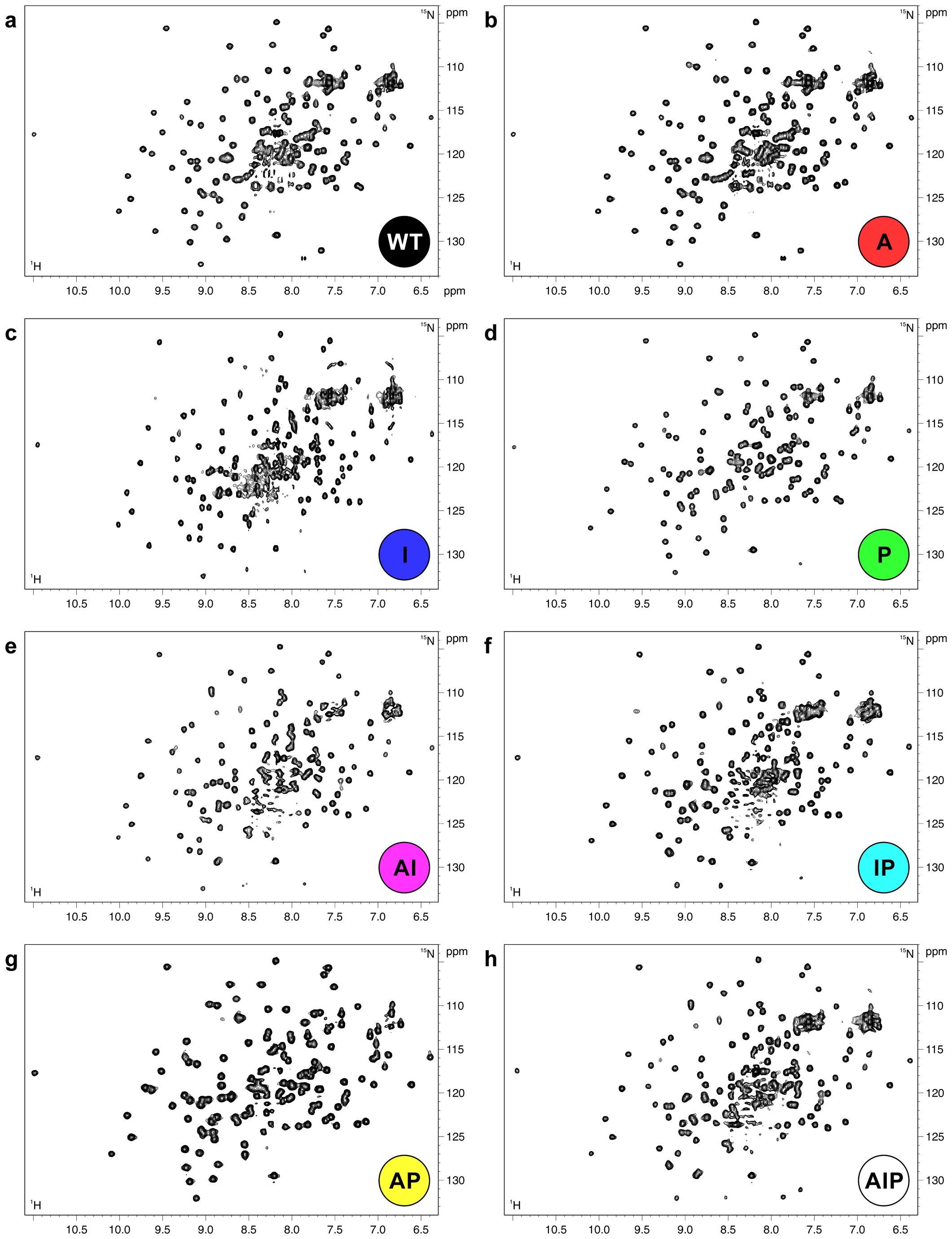


**Supplementary Figure 2.** ^1^H-^15^N SOFAST-HMQC spectra of human cell lysates containing U-^15^N labelled WT PFN1 (a), single-type mutants (b-d), double-type mutants (e-g) and triple-type mutant (h). From inspection of the amide chemical shifts, the fold of all mutant proteins (b-h) appear unchanged with respect to the WT (a), as most crosspeaks are not perturbed by the mutations. The labels are color-coded as in the diagram shown in Figure 1d.

**
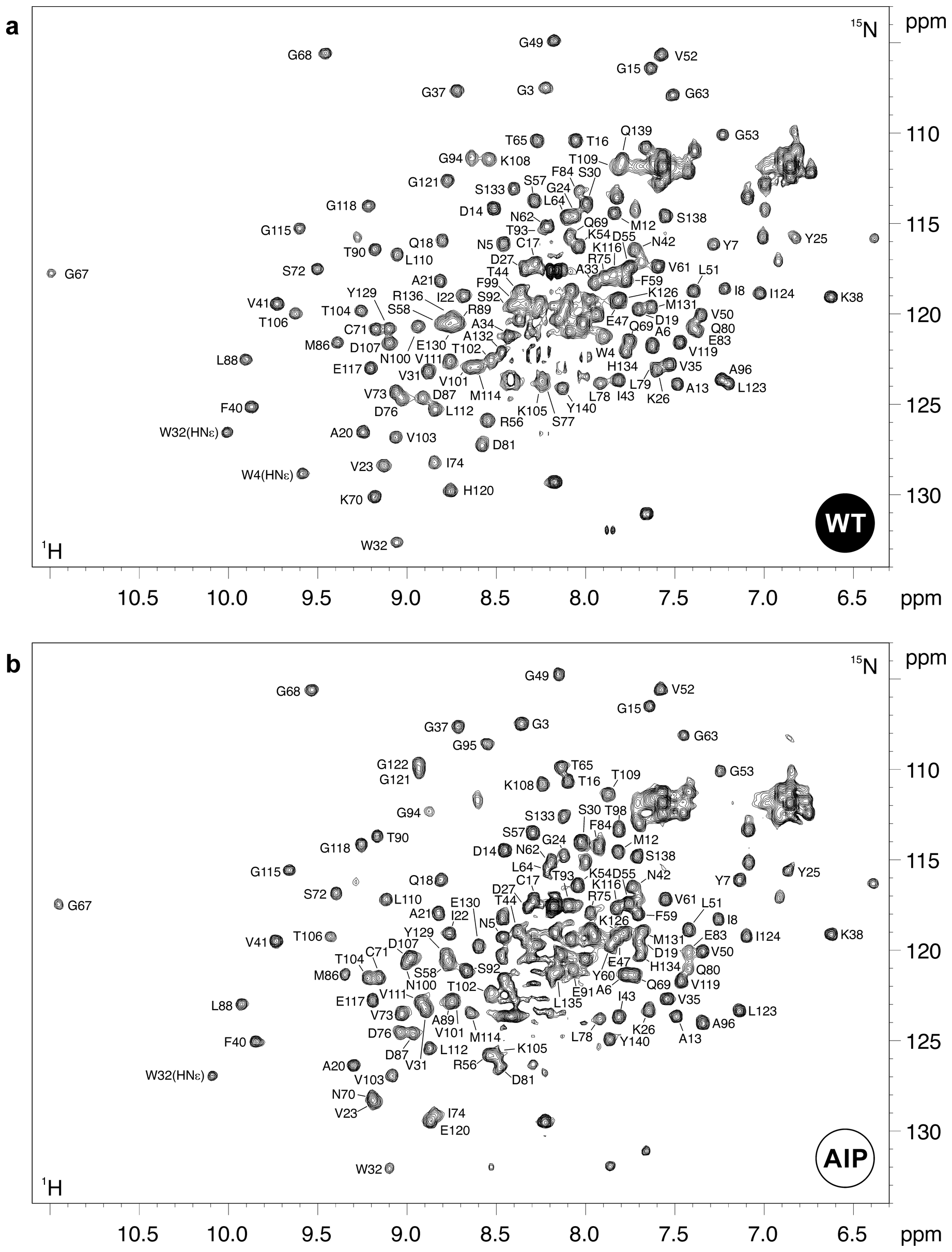
**

**Supplementary Figure 3.** ^1^H-^15^N SOFAST-HMQC spectra of human cell lysates containing U-^15^N labelled WT PFN1 (a) and “AIP” mutant (b). Sequence-specific resonance assignments are indicated.


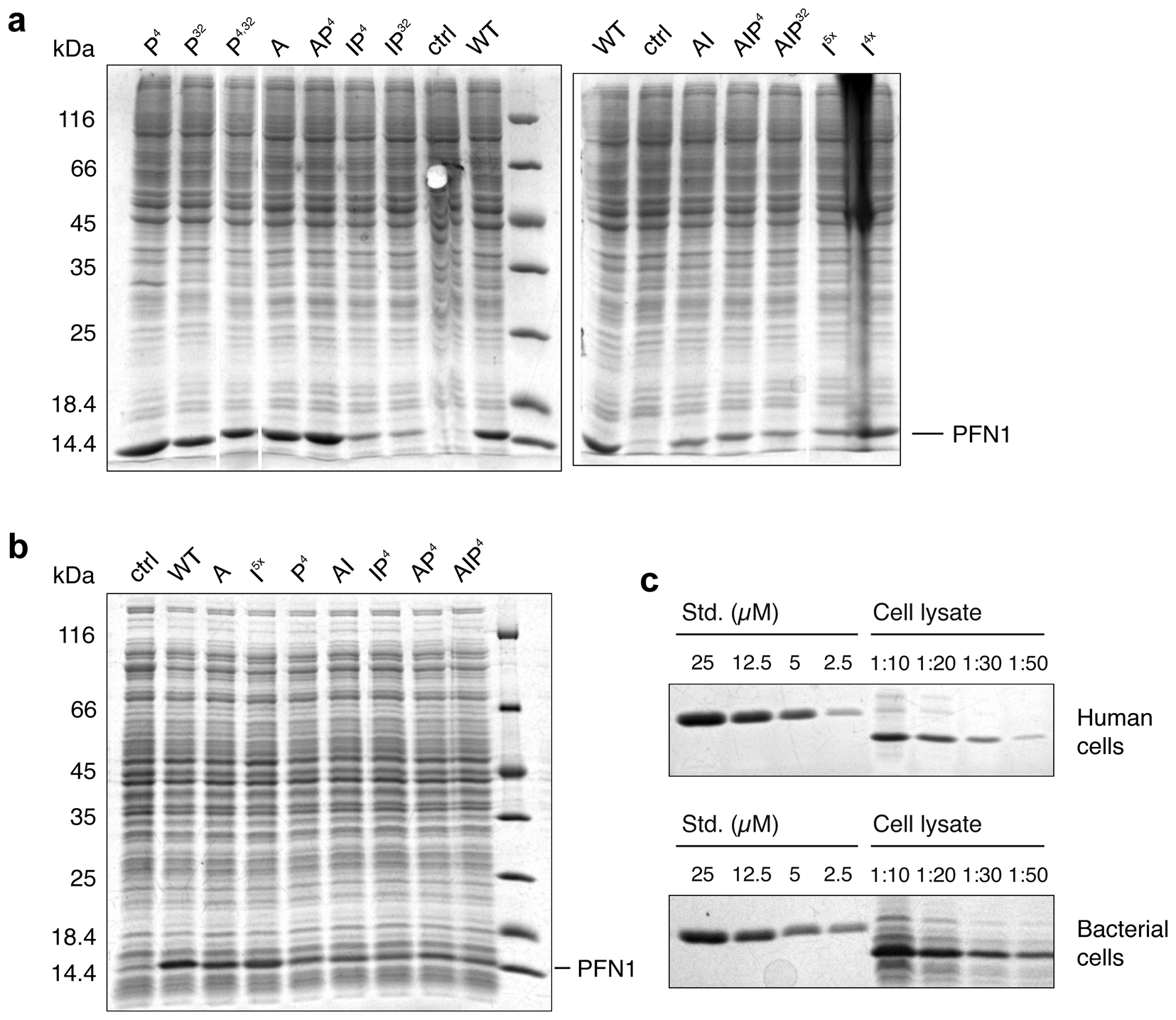


**Supplementary Figure 4.** Coomassie-stained SDS-PAGEs of human (a) and bacterial (b) cleared cell lysates obtained from cells overexpressing WT PFN1 and different PFN1 mutants. The mutant labels follow the scheme reported in Table 1. (c) Expression level of WT PFN1 in human and bacterial cells estimated by Coomassie-stained SDS-PAGE with a standard sample of pure His-PFN1 at known concentration. Calculated WT PFN1 expression levels are 130 ± 30 µM (s.d.) in human cells and 300 ± 100 µM (s.d.) in bacterial cells.


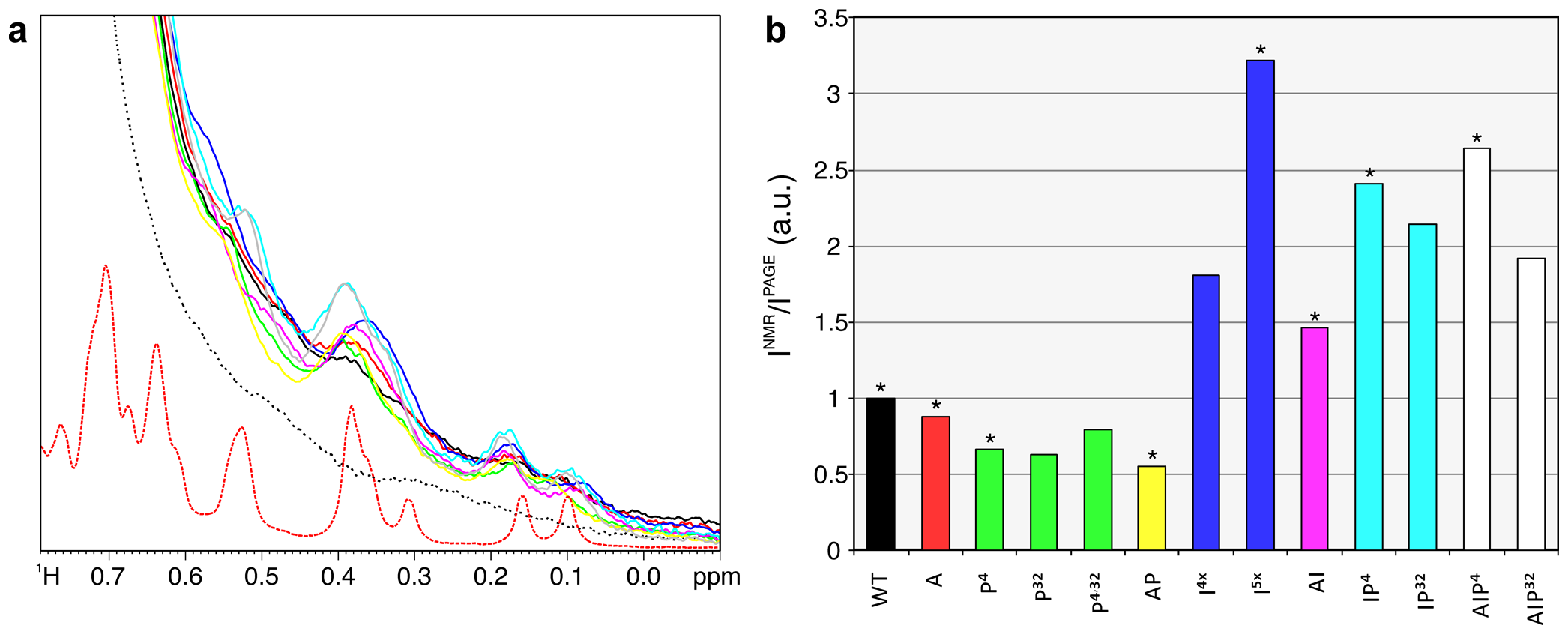


**Supplementary Figure 5.** (a) Overlay of the aliphatic region of the ^1^H NMR spectra of samples of human cells expressing different PFN1 mutants, color-coded as in Figure 1d (except “AIP” which is shown in light gray). The signal intensity is proportional to the amount of PFN1 visible to ^1^H solution NMR. The spectra were scaled based on ^1^H signals arising from other cellular components (not shown). A ^1^H NMR spectrum of human cells transfected with an empty vector (dotted black line) was used as blank for calculating the signal intensity. The blank spectrum was subtracted from each in-cell NMR spectrum, and the residual ^1^H signal was integrated over the aliphatic region (between 0.76 and 0 ppm). The spectrum of pure WT PFN1 is shown as reference (dashed red line). (b) Ratios between the integrated ^1^H signal intensity of the ^1^H NMR spectra and the normalized band intensity of a Coomassie-stained SDS-PAGE of each PFN1 mutant (Supplementary Fig. 3a), which is proportional to the total level of protein. For some types of mutants, more combinations were analyzed (the labels follow the scheme reported in Table 1). The mutants for which ^1^H-^15^N NMR spectra were also recorded are indicated with an asterisk. The color code is the same as in Figure 1d.

**
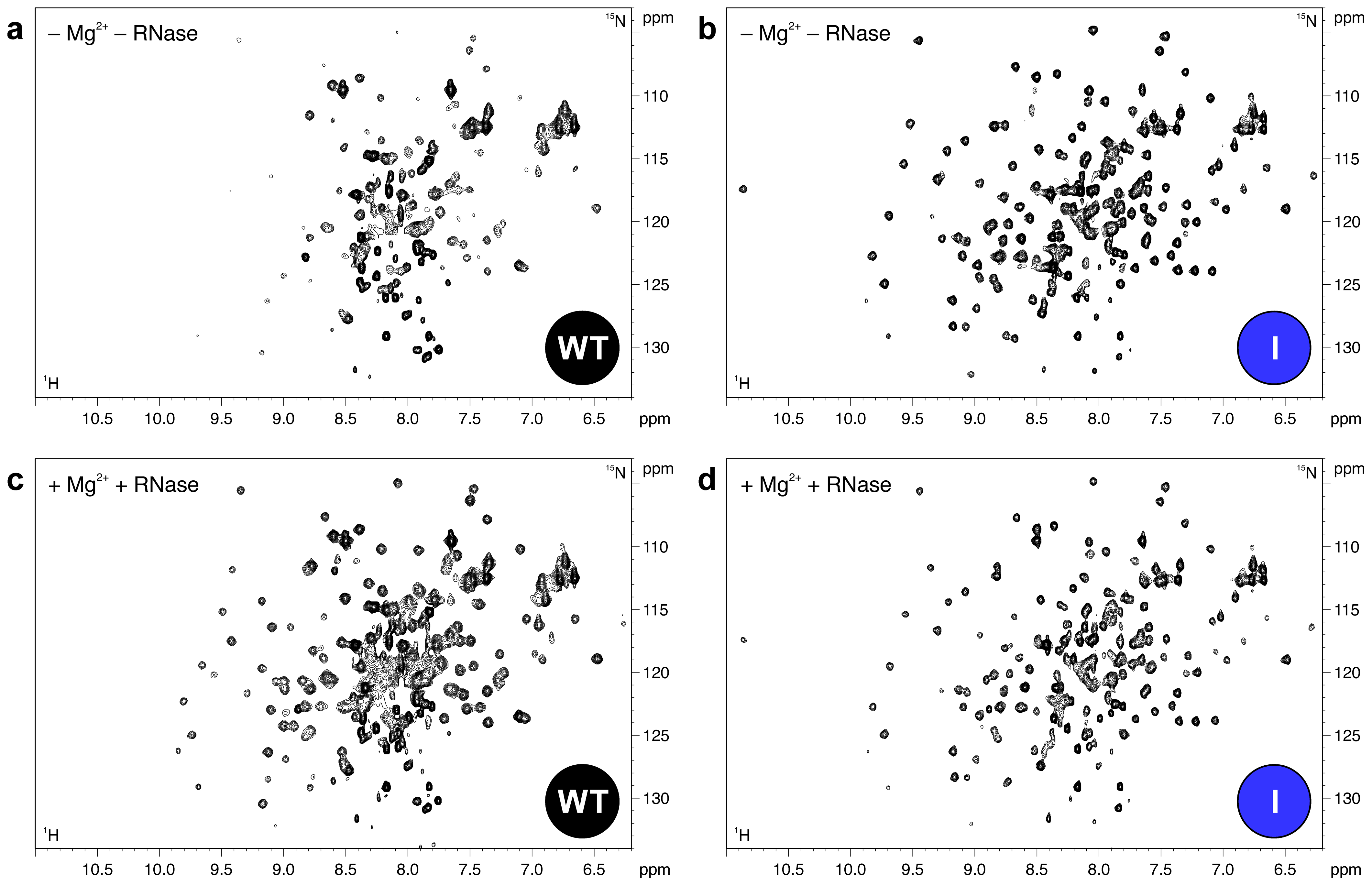
**

**Supplementary Figure 6.** ^1^H-^15^N SOFAST-HMQC spectra of bacterial cell lysates containing U-^15^N labelled WT PFN1 (a,c) and “I” mutant (b,d) at 296 K. (a,b) Untreated lysates; (c,d) lysates after incubation with RNase A and addition of Mg^2+^. The labels are color-coded as in Figure 1d.
